# Household malaria knowledge and its association with bednet ownership in settings without large–scale distribution programs: Evidence from rural Madagascar

**DOI:** 10.7189/jogh.04.010401

**Published:** 2014-06

**Authors:** Paul J. Krezanoski, Alexander C. Tsai, Davidson H. Hamer, Alison B. Comfort, David R. Bangsberg

**Affiliations:** 1 Department of Medicine, Massachusetts General Hospital, Boston, Massachusetts, USA; 2 Department of Pediatrics, Massachusetts General Hospital, Boston, Massachusetts, USA; 3 Center for Global Health, Massachusetts General Hospital, Boston, Massachusetts, USA; 4 Department of Psychiatry, Massachusetts General Hospital, Boston, Massachusetts, USA; 5 Harvard Medical School, Boston, Massachusetts, USA; 6 Center for Global Health and Development, Boston University, Boston, Massachusetts, USA; 7 Department of International Health, Boston University School of Public Health, Boston, Massachusetts, USA; 8 Zambia Center For Applied Health Research and Development (ZCAHRD), Lusaka, Zambia; 9 Section of Infectious Diseases, Department of Medicine, Boston University Medical Center, Boston, Massachusetts, USA; 10 Abt Associates, International Health Division, Cambridge, Massachusetts, USA; 11 Harvard School of Public Health, Boston, Massachusetts, USA; 12 Ragon Institute of MGH, MIT, and Harvard, Charlestown, Massachusetts, USA; 13 Faculty of Medicine, Mbarara University of Science and Technology, Mbarara, Uganda

## Abstract

**Background:**

Insecticide–treated bednets are effective at preventing malaria. This study focuses on household–level factors that are associated with bednet ownership in a rural area of Madagascar which had not been a recipient of large–scale ITN distribution.

**Methods:**

Data were gathered on individual and household characteristics, malaria knowledge, household assets and bednet ownership. Principal components analysis was used to construct both a wealth index based on household assets and a malaria knowledge index based on responses to questions about malaria. Bivariate and multivariate regressions were used to determine predictors of household bednet ownership and malaria knowledge.

**Results:**

Forty–seven of 560 households (8.4%) owned a bednet. In multivariate analysis, higher level of malaria knowledge among household members was the only variable significantly associated with bednet ownership (odds ratio 3.72, *P* < 0.001). Among respondents, predictors of higher malaria knowledge included higher education levels, female sex and reporting fever as the most frequent or dangerous illness in the community. Household wealth was not a significant predictor of bednet ownership or respondent malaria knowledge.

**Conclusion:**

In this setting of limited supply of affordable bednets, malaria knowledge was associated with an increased probability of household bednet ownership. Further studies should determine how such malaria knowledge evolves and if malaria–specific education programs could help overcome the barriers to bednet ownership among at–risk households living outside the reach of large–scale bednet distribution programs.

Malaria is a leading cause of mortality and morbidity in sub–Saharan Africa, accounting for over one million deaths each year and 600 000–800 000 deaths among children less than five years of age [1,2]. Malaria is a significant health problem in Madagascar, representing a significant burden for the health system. Malaria–related illness makes up 16% of all outpatient visits and is the leading cause of child mortality, killing nearly 20 000 children under five years of age every year [3].

Insecticide–treated bednets (ITNs) are one of the most effective tools for preventing malaria [4,5]. ITNs are estimated to be as cost–effective as the measles vaccination [6] and ITN ownership is associated with an 18%–23% decrease in child mortality in sub–Saharan African households [7]. According to the World Health Organization (WHO), large–scale distribution programs in sub–Saharan Africa have improved access to ITNs in recent years, with the percentage of households owning at least one ITN rising from an average of 3% in 2000 to 53% in 2012 [8]. Nevertheless, ITN coverage continues to lag behind the Roll Back Malaria Partnership goal of 80% ITN coverage of under five-year-old children by 2010. Indeed, in Madagascar, only 46% percent of children under five were sleeping under ITNs based on 2006–2010 estimates [9].

Understanding what factors predict household ownership and use of nets is important for improving policies and programs to increase ITN coverage. Most recent studies of the predictors of bednet ownership and use have taken place in the setting of large distribution campaigns. Fewer studies have looked at the predictors of bednet ownership and use outside of subsidized distribution programs or outside of controlled trials [10–13]. This is of particular interest because households that do not contain members who are targeted for subsidized distribution programs, such as children under 5 years or pregnant women, continue to encounter a relatively scarce supply of quality and affordable ITNs [14,15].

We undertook this study in order to identify household–level factors that are associated with bednet ownership in a rural area of Madagascar. We were interested in why some households seek out bednets even when they are not provided for free or as part of large–scale programs. We also investigated predictors of high levels of malaria knowledge to further our understanding of the generic origins of household demand for ITNs.

## METHODS

This study is a secondary analysis of baseline data from a previously reported cluster randomized–controlled trial. That study was a comparison of the effects of household–level incentives on bednet ownership and use in rural villages in the Ambalavao district of Madagascar. Full details of the study design have been published previously [16]. In brief, 20 villages within 5 km of Ambalavao town were included and all households within each village were eligible for participation. The entire country of Madagascar is considered to be at risk for malaria. The Ambalavao district is located in the southern highlands in the Haute Matsiatra region and experiences stable year–round malaria transmission, with the majority of cases occurring during the rainy season from January to April. The study began in 2007, with no recent free nor subsidized bednet distribution programs in the area. Bednets were available only at specialized pharmacies and private shops in Ambalavao town. Thus, this study was implemented in a context similar to many other sub–Saharan African countries where bednets are available in scarce supply and/or with significant cost barriers to ownership.

A baseline survey was performed to collect demographic information about the households. The survey respondent was preferably the head of household if available at the time of the home visit, otherwise the respondent was another adult in the household. Information recorded included the respondent’s relation to the head of household, age, gender, education level, perceptions of malaria risk, malaria knowledge, and household assets, fuel and water sources, self–reported bednet ownership, visual confirmation of whether a bednet was mounted above a sleeping surface in the household, recent fevers and fever–related deaths in the household. The Malagasy term *tazo moka*, literally “fever from mosquitoes,” was used in all questions about malaria, unless otherwise stated.

The primary outcome of interest was household bednet ownership at the time of the baseline survey. A secondary outcome was the survey respondent’s malaria knowledge as defined by the malaria knowledge index detailed below. Bednet ownership was based on self–reported ownership by the survey respondent; ownership was not verified by the surveyor. Bednet use was not used as an outcome because of the inadequate variation for in–depth analysis due to the small sample of households that owned bednets.

The following variables were examined as potential predictors of bednet ownership: the age, gender and years of formal schooling of the household head, number of household members, occurrence of a febrile illness within the household in the last month, number of children under five years of age, number of pregnant women, use of an open water source, distance to water source, household wealth quintile (detailed below), the survey respondent’s report of fever as the most dangerous or most common illness in the community and the respondent’s malaria knowledge index (detailed below).

A wealth index was constructed by applying principal components analysis to twenty–seven binary variables representing household assets including goods, livestock, and housing characteristics such as roof and flooring materials, and number of rooms and beds [17]. The first principal component was extracted and designated as the wealth index. Only the number of rooms and beds in the household were adjusted for the number of household inhabitants [18]. Per convention, the wealth index was categorized into quintiles for analysis.

Similar to the wealth index described above, we constructed a malaria knowledge index by applying principal components analysis to the responses to twelve questions about the mechanism of transmission of malaria, malaria symptoms, knowledge of greater severity of malaria in children and pregnant women, knowledge of malaria seasons and means of protection against malaria. The first principal component was extracted and designated as the malaria knowledge index. Given the distribution of the malaria knowledge scores (see below), participants were categorized into 2 groups representing low and high levels of malaria knowledge.

All analyses were performed using Stata 10 (StataCorp, College Station, Tex., USA). First, we used bivariate logistic regression models for each independent variable with household bednet ownership as the dependent variable. Then, we conducted a multivariate logistic regression including all independent variables which had a significant predictive value with a *P* < 0.25 in the bivariate analysis [19]. The steps above for the bivariate and multivariate regressions were repeated with an analysis looking at the determinants of malaria knowledge, with the malaria knowledge index as a dependent variable and independent variables capturing household and respondent characteristics. Finally, since the survey respondents were not always heads of households or other “decision makers” in the household, we performed confirmatory sub–analyses restricting the sample to only survey respondents who were 1) heads of households, 2) wives of heads of households or 3) household decision makers, defined as either heads of households or wives of heads of households.

As noted in the original study [16], “Ethical clearance for this study was provided by the Boston University Medical Campus Institutional Review Board. Additional administrative approval was provided by the mayor of the town of Ambalavao, responsible for the villages in the district, and the Medicin Inspecteur of the Ambalavao health district, the local official in charge of all health–related activities in the district. Additionally, the chiefs of each village gave their approval for the study to take place in their village. Study participants provided verbal consent at the time of the surveys and coupons for the free ITNs were provided to all households in the study villages irrespective of whether or not they consented to participate in the study.” In addition, the specific analysis described in this manuscript was reviewed and approved by the Partners Human Research Committee.

## RESULTS

Data were collected from 560 households containing 2881 individuals in 20 villages (**Table 1**). A majority of households (n = 346, 62%) had at least one child under five years old and 29 (5.2%) had a pregnant woman residing there. Thirty–three percent of households (186) reported having at least one household member with a fever in the preceding month. Among the survey respondents, 254 (45%) were the heads of their households and 250 (45%) were the wives of the household heads. Most respondents were female (452, 81%) and had an average age of 39 years. Respondents averaged approximately 5 years of education and 69 (12%) respondents had never attended school.

**Table 1:** Village, household and individual respondent characteristics

Variable	
Village characteristics (n = 20 villages)	
Households per village (mean ± SD)	28.0 ± 14.7 (range 9–61)
Households using open water source (n [%])	371 (66.3%)
Village distance to open water source (meters) (mean ± SD)	8.9 ± 8.6 (range 0–40)
**Household characteristics** **(n = 560 households)**	
Total individuals in study households	2881
Members per household (mean ± SD)	5.1 ± 2.7 (range 1–20)
Female head of household (n [%])	150 (26.8%)
Men per household (mean ± SD)	2.4 ± 1.7
Women per household (mean ± SD)	2.7 ± 1.7
Children under 5 per household (mean ± SD)	0.94 ± 0.90 (range 0–5
Households with at least one child under 5 (n [%])	346 (61.8%)
Pregnant women per household (mean ± SD)	0.05 ± 0.23 (range:0–2)
Households with at least one pregnant woman (n [%])	34 (5.2%)
Households reporting member with fever in last month (n [%])	186 (33.2%)
Households reporting death last year due to fever (n [%])	1 (0.2%)
**Selected household asset characteristics (n = 560 households)**	
Thatch roofing (n, %)	440 (78.6%)
Dirt flooring (n, %)	504 (90.0%)
Dirt/mud walls (n, %)	555 (99.1%)
Charcoal for main cooking fuel (n, %)	534 (95.4%)
Number of cattle (mean ± SD)	1.4 ± 2.8 (range 0–27)
Number of chickens (mean ± SD)	3.7 ± 9.2 (range 0–100)
Own at least one… (n, %)	
…radio	424 (75.7%)
…bicycle	149 (26.6%)
…cellphone	21 (3.8%)
…cattle drawn cart	40 (7.1%)
… motorcycle/automobile	0 (0%)
**Individual respondent characteristics** **(n = 560 individuals)**	
Gender – female (n, %)	452 (80.7%)
Age (mean ± SD)	38.7 ± 16.3 (range 14–96)
Married (n, %)	368 (65.7%)
Relation to head of household (n, %):	
wife	254 (45.4%)
household head	250 (44.6%)
child	44 (7.9%)
other (parent, sibling, grandchild)	12 (2.1%)
Number of children (mean ± SD)	3.5 ± 2.8 (range 0–15)
Years of education (mean ± SD)	4.9 ± 2.9
Number of years in school (n, %)	
0	69 (12.3%)
1–4	183 (32.7%)
5–8	224 (40.0%)
9–12+	84 (15.0%)
Self–reported literacy (n, %)	487 (87.0%)

SD – standard deviation

Eighty–two percent of respondents (n = 489) identified fever as the most common illness in their communities and 294 respondents (53%) reported fever as the most dangerous illness (**Table 2**). Seventy–three percent identified mosquitoes as the mechanism for acquiring fevers and 44% listed bednets and avoidance of mosquitoes as a means of protection against malaria. Most respondents recognized that malaria is more severe in children (67%) and pregnant women (69%). Eighty–one percent of respondents reported that malaria is most common when the weather is wet and 87% thought malaria was most common when it is cold.

**Table 2:** Beliefs and perceptions of malaria (n = 560)

Perception/belief	No. (%)
**Perceptions of malaria:**	
Fever as most **frequent** illness in village	489 (82.3)
Fever as most **dangerous** illness in village	294 (52.5)
**Mechanism of acquiring malaria:**	
Mosquitoes	407 (72.7)
Getting chilled/overheated	48 (8.6)
Poor hygiene/unkempt household	45 (8.1)
Bad food/water	15 (2.6)
Unripe fruits	13 (2.4)
Other (fatigue, rats, etc)	9 (1.6)
Unsure	23 (3.9)
**Symptoms of malaria:**	
Chills/rigors	249 (44.4)
Headache	228(40.8)
Listless	29 (5.1)
Vomiting	16 (2.8)
Agitation	9 (1.6)
Seizures/shaking	2 (0.3)
Other (coughing, trouble breathing, etc)	15 (2.9)
Unsure	12 (2.1)
**Protection against malaria:**	
Use bednet/avoid mosquito bites	246 (44.0)
Use natural medicines	108 (19.3)
Keep household clean	85 (15.1)
Take medications	62 (11.1)
Visit hospital/doctor	13 (2.3)
Avoid unripe fruits	12 (2.1)
Stay warm	9 (1.6)
Other (boil water, get vaccinated, etc)	11 (2.1)
Unsure	14 (2.6)
**Severity pairings:**	
Malaria is **more of a risk** in…	
…men	52 (9.3)
…women	(321 57.3)
…same	187 (33.4)
Malaria is **more of a risk** in…	
…adult	6 (1.1)
…child	376 (67.1)
…same	178 (31.8)
Malaria is **more of a risk** in…	
…non–pregnant women	5 (0.9)
…pregnant woman	385 (68.8)
…same	170 (30.4)
**Frequency pairings:**	
Malaria is **most common** in…	
…wet season	452 (80.7)
…dry season	100 (17.9)
…same	8 (1.4)
Malaria is **most common** in…	
…cold season	489 (87.3)
…warm season	62 (11.1)
…same	9 (1.6)

**Figure 1:**
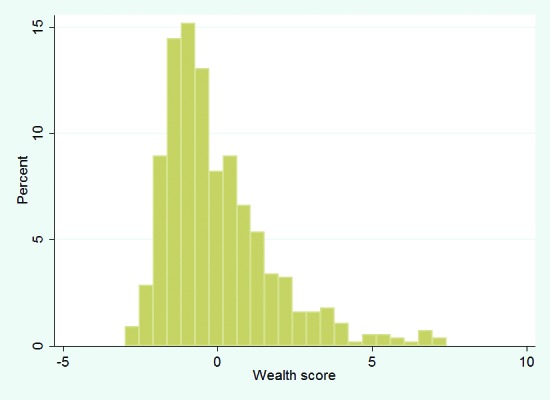
Distribution of wealth index for principal component analysis of household assets.

**Figure 2:**
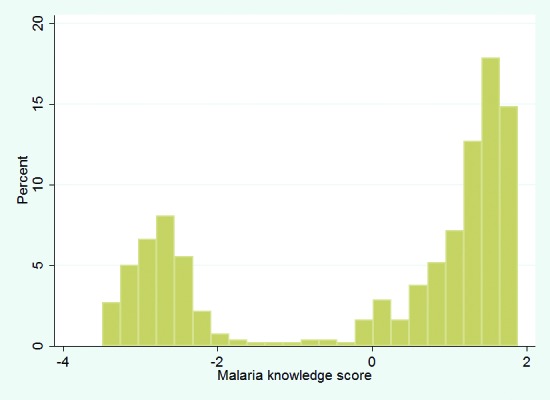
Distribution of knowledge index for principal component analysis of respondent malaria knowledge.

The wealth index had a normal distribution with a rightward skew (**Figure 1**), while the distribution of malaria knowledge in this population, as defined by our knowledge index, showed a bimodal pattern (**Figure 2**).

At baseline, 47 households (8.4%) owned a bednet and 34 (6.1%) had it mounted over a sleeping area as observed by the surveyor (**Table 3**). Most households had paid for their bednet, with 13 paying 1000 Ar (Malagasy Ariary; US$ 0.63), 27 households paying 3000 Ar (US$ 1.90) and the source was unavailable for seven bednets. Overall, 25 of 524 (4.8%) children under 5 years of age were reported to have slept under a bednet the night before. Among households in which a bednet was observed to be mounted over a sleeping area, 25 out of 33 (76%) of children under 5 were reported to have slept under the bednet the previous night. One of 30 households (3.3%) with pregnant women reported that the pregnant woman slept under a bednet the night before.

**Table 3:** Household (n = 560) bednet ownership characteristics

Characteristics	No (%)
Report net ownership	47 (8.4)
Observed net mounted	34 (6.1)
Number of nets owned:	
one	44/47 (93.6)
two	3/47 (6.4)
Age of net (years):	
≤1	23/47 (27.7)
2–4	11/47 (23.4)
≥5	5/47 (10.6)
unsure	8/47 (17.0)
Reports child under 5 y using a net the night before	25/524 (4.8)
…if household owns bednet	25/47 (53.2)
…if bednet is mounted	25/33 (75.8)
Reports pregnant women using a net the night before	1/30 (3.3)
…if household owns bednet	1/1 (100.0)
…if bednet is mounted	1/1 (100.0)

The results from the bivariate analysis show that malaria knowledge, household wealth, household size, households reporting a fever during the previous month were all associated with bednet ownership (**Table 4**). In the multivariate analysis, a higher level of malaria knowledge was the only variable significantly associated with household bednet ownership (OR 3.72, 95% confidence interval (CI) 1.83–7.55%, *P* < 0.001). Evaluated at the mean of the other covariates, a household with a survey respondent with less malaria knowledge had a 3.4% likelihood of owning a bednet (95% CI 1.9–6.2%) vs 11.6% (95% CI: 8.2%–16.1%) in households with respondents with higher levels of malaria knowledge. A similar strong association between malaria knowledge and bednet ownership was the only significant finding in multiple sub–analyses of the sample, including survey respondents who were heads of households only, wives of heads of households only and household “decision makers”, ie, heads of households or wives of heads of households (Online Supplementary Document(Online Supplementary Document)). Restricting the sample to respondents who were heads of households only showed an even stronger association with bednet ownership (OR 5.82, 95% CI 1.09–30.84, *P* = 0.038).

**Table 4:** Significant correlates of household bednet ownership (n = 560 observations)

Variable	Bivariate analysis		Multivariate analysis	
	OR (95% CI)	P value	OR (95% CI)	P value
Head of household (HH) characteristics:*				
Age of HH	1.01 (0.99–1.04)	0.352	–	–
Gender of HH	0.66 (0.21–2.11)	0.483	–	–
Education level of HH	1.08 (0.90–1.30)	0.413	–	–
**Household characteristics:**				
Number of HH members	1.12 (1.02–1.24)	0.019	1.09 (0.98–1.22)	0.125
Reported fever in last month	1.70 (0.93–3.12)	0.084	1.49 (0.79–2.79)	0.217
Number of children under 5 years	1.12 (0.81–1.54)	0.497	–	–
Number of pregnant women	1.21 (0.37–3.96)	0.753	–	–
Open water source	0.99 (0.52–1.85)	0.965	–	–
Distance to water source (minutes walk)	1.00 (0.97–1.03)	0.976	–	–
**Wealth index **(quintile relative to lowest wealth quintile):				
Second	0.95 (0.33–2.73)	0.930	0.73 (0.25–2.15)	0.562
Third	0.64 (0.20–2.01)	0.441	0.53 (0.16–1.71)	0.284
Fourth	1.34 (0.51–3.52)	0.558	1.00 (0.36–2.79)	0.999
Fifth	2.44 (1.01–5.90)	0.048	1.84 (0.69–4.95)	0.226
**Respondent perception/knowledge of malaria:**				
Reports fever most frequent illness in village	0.68 (0.31–1.53)	0.352	–	–
Reports fever most dangerous illness in village	1.24 (0.68–2.27)	0.479	–	–
Malaria knowledge index	3.61 (1.80–7.24)	<0.001	3.72 (1.83–7.55)	<0.001

CI – confidence interval

*n = 256 because of missing variables.

Significant independent correlates with a higher malaria knowledge index score included the respondent’s perception that malaria was the most frequent or most dangerous illness in the community, female gender, being married, education level of the respondent and whether the household reported a fever in the previous month (**Table 5**). In the multivariate analysis, high levels of malaria knowledge were correlated with both the respondent’s education level (OR 1.11, 95% CI 1.04–1.18, *P* = 0.001) and the respondent being a female (OR 1.77, 95% CI 1.12–2.79, *P* = 0.015). Additionally, reporting a fever as the most frequent (OR 2.34, 95% CI 1.00–5.47, *P* = 0.049) or most dangerous illness in the community (OR 1.87, 95% CI 1.09–3.22, *P* = 0.023) was associated with higher respondent malaria knowledge. However, fevers reported in the household in the last month were not predictive of higher malaria knowledge.

**Table 5:** Correlates of higher levels of malaria knowledge (n = 560 observations)

	Bivariate analysis		Multivariate analysis	
**Variable**	OR (95% CI)	P value	OR (95% CI)	P value
**Respondent characteristics and perceptions:**				
Age of respondent	1.00 (0.99–1.01)	0.560	–	–
Female gender	2.02 (1.31–3.12)	0.001	1.77 (1.12–2.79)	0.015
Marriage status	1.29 (0.91–1.83)	0.155	1.21 (0.83–1.75)	0.315
Number of children	1.00 (0.95–1.06)	0.916	–	–
Pregnancy status	1.21 (0.51–2.85)	0.430	–	–
Education level	1.16 (1.05–1.18)	<0.001	1.11 (1.04–1.18)	0.001
Reports fever most common illness	2.90 (1.68–5.00)	<0.001	2.38 (1.35–4.19)	0.003
Reports fever most dangerous illness	2.12 (1.52–2.98)	<0.001	1.81 (1.27–2.59)	0.001
**Household characteristics:**				
Number of inhabitants	0.99 (0.93–1.05)	0.673		
Reported fever in last month	1.47 (1.03–2.10)	0.032	1.27 (0.87–1.86)	0.210
Number of children under 5 y	1.11 (0.92–1.34)	0.261	–	
Number of pregnant women	1.30 (0.64–2.67)	0.470	–	
Open water source	0.84 (0.59–1.19)	0.326	–	
Distance to water source	1.00 (0.98–1.02)	0.654	–	
Wealth index in greatest 20%	0.87 (0.51–1.46)	0.593	–	
Wealth index in lowest 20%	0.92 (0.55–1.54)	0.746	–	

CI – confidence interval

## DISCUSSION

In this cross–sectional analysis of data from 560 households in rural Madagascar without access to ITNs as part of a large–scale ITN distribution program, malaria knowledge was independently and highly associated with bednet ownership. Our multivariate model demonstrated that households with high levels of malaria knowledge, derived from a principal components analysis, were nearly 3.5 times more likely to own a bednet compared to households with low levels of malaria knowledge, even after adjusting for potential confounders such as years of education and household wealth.

Multiple methods for categorizing and quantifying malaria knowledge have been used previously [10,11,15,20–22]. Most studies have used variations on a scoring system which provides points for correct answers to questions related to malaria knowledge and then categorizes respondents based on their scores. Hwang et al. in a study in Ethiopia [23] considered the use of principal components analysis for quantification of malaria knowledge but instead used a dichotomized scoring system comparing groups with no malaria knowledge (zero correct answers) to any correct malaria knowledge (≥1 correct answer). The authors justified such an approach because their result using the dichotomized score was equivalent to the principal components approach and was easier to interpret. Malaria knowledge in this Ethiopian cohort, however, was quite limited and only 4 knowledge questions were posed. In our study, participants answered correctly a mean of 7.1 out of 12 questions (standard deviation = 1.8) and there were no women who answered incorrectly to all. The bimodal distribution of **Figure 2** supports our classification of malaria knowledge in 2 groups in our population, but the breadth of the questions we posed adds richness to our knowledge index.

Multiple village–, household– and individual–level characteristics have been associated with bednet ownership and use in a variety of bednet distribution settings, including education level, socio–economic status, perceptions of malaria risk and malaria knowledge. While some studies have found an increase in bednet ownership and use among those more knowledgeable about malaria [24,25], other studies have found little or no association [26,27].

Despite the association between malaria knowledge and bednet ownership in our study, an analysis of the individual components of our malaria knowledge index suggests important gaps in malaria knowledge in the sample. Only 73% of villagers replied that the primary means of acquiring malaria is from mosquitoes, 44% identified bednets as a means to protect against malarial fevers and only 67% of respondents identified children and 69% identified pregnant women as more vulnerable to malaria compared to adults or non–pregnant women, respectively. Finally, the prevailing view was that malaria was most common in the colder season, which is not typical for malaria in either Madagascar or in other sub–Saharan African settings and may represent overlap with perceptions of other causes of fevers.

Incorrect responses to questions about malaria are common in the literature, with heterogeneous levels of malaria knowledge across geographical and cultural settings. For example, a study in Tanzania among pregnant women in 2004 found nearly the opposite results: only 35% identified mosquitoes as the means of transmission of malaria and 91% reported bednets as a primary means of protection [10]. Our findings suggest that knowledge–based interventions should continue to be explored as a means to improve malaria bednet uptake, but the exact components of those interventions should account for the particular gaps in knowledge in a population given variations across, and potentially within, countries.

In addition to gaps in specific malaria knowledge, there were important gaps in optimal prevention behaviors. Only 8.2% of households owned a bednet. In addition, the presence of children under 5 years of age or pregnant women in the household was not associated with increased bednet ownership and only 53% of children under 5 years of age slept under a bednet the night before, even when the household owned a bednet (**Table 3**).

Higher wealth status, when categorized by quintile, was not associated with bednet ownership. This may seem unexpected in a setting where bednets are expensive relative to individuals’ average income. Numerous studies, including an unpublished study from the region (Comfort and Krezanoski, in preparation), have shown that the price of bednets significantly affects the likelihood of households owning a bednet. Nevertheless, the absolute wealth of this population may be so low as to minimize relative differences in wealth in terms of ability to afford a bednet. In support of this interpretation, an examination of the wealth–index primary component scores (**Figure 1**) shows that the distribution is rightward skewed indicating that the majority of households are concentrated around a lower wealth level, with a small proportion of relatively affluent households.

In terms of assessing malaria knowledge, principal components analysis has the advantage of providing a means of comparison that is independent of the specific components making up that knowledge score. Principal components approaches also mitigate the problem of equally weighting responses about malaria knowledge, where, for example, sophisticated knowledge components, such as knowing that malaria can result in pregnancy loss, are given equal weight as basic components, such as knowing that malaria is transmitted by mosquitoes. Finally, our knowledge index measures each respondent relative to the study sample, allowing us to investigate how variations in malaria knowledge distinguish households from each other within a particular malaria–risk context.

Identification of malaria knowledge as a predictor of malaria prevention behaviors suggests a focus on malaria–specific education as a means of increasing bednet coverage even in settings without large scale distribution and subsidization of bednets [28,29]. However, questions remain as to the origins of the malaria knowledge measured in this study which appears correlated with household bednet ownership. In this study, respondents with higher levels of malaria knowledge had more education, confirming a correlation between formal schooling and health learning that has been found in previous studies [25,26]. The findings of higher malaria knowledge among respondents reporting that malaria is the most frequent or the most dangerous illness in the community and the association of higher malaria knowledge with female gender may be a marker of more frequent exposures to educational interventions delivered during prior treatment episodes for the individual or members of their family (especially children). This may be the result of health education occurring during health facility visits and reinforces the importance of interventions delivered during the evaluation and treatment of malaria episodes as a means of improving malaria prevention. Living in a higher wealth household was not associated with higher malaria knowledge, contrary to what may be expected through better access to formal education or exposure through media (radio, etc.) to health messages. Like bednet ownership, malaria knowledge in this population appears to be independent of relative household affluence.

Interpretations of these findings are subject to five limitations. First, we did not have an adequate sample size to examine the determinants of bednet use as opposed to ownership. The former is a much more relevant indicator for malaria prevention. Second, households that did not own nets were not asked if they desired a bednet nor were they asked about the perceived barriers to bednet ownership, including cost. Third, we assessed malaria knowledge only among one individual in the household (the survey respondent) and this respondent may or may not have equal impact on bednet ownership in the family, ie, less senior members. Nevertheless our main findings were consistent when restricting the analyses to the main decision making individuals (heads of households and their wives) within the household. Fourth, the local term for malaria, *tazo moka* (“fever from mosquitoes”), can be confused with *tazo* (general term for “fever”), thus there could have been misclassification of fevers not attributable to malaria. Finally, our survey did not ask participants about the primary source of their malaria knowledge nor did we perform qualitative studies to further explore the characteristics of their malaria knowledge. This information would help us determine whether malaria knowledge is coming from formal education, interactions with health workers or community health workers, media or from other sources.

## CONCLUSIONS

In summary, in this secondary analysis of baseline data from 560 individuals participating in a randomized controlled trial in a setting without widespread access to bednets in rural Madagascar, we found that household knowledge of malaria was independently associated with an increased probability of bednet ownership. Higher levels of malaria knowledge were associated with reported concern about malaria as a common and dangerous illness in the community as well as higher education and female gender, but were not associated with the wealth of the household. Further studies are warranted to elucidate the origins of better malaria knowledge and to determine how such knowledge may be best operationalized, possibly through education programs, to overcome the barriers to bednet ownership among at–risk households living outside the reach of large–scale distribution programs.
