# Supplementary material for: Household malaria knowledge and its association with bednet ownership in settings without large–scale distribution programs: Evidence from rural Madagascar
Source: J Glob Health. 2014 Jun;4(1):010401. doi: 10.7189/jogh.04.010401 (PMC4073249; doi:10.7189/jogh.04.010401)
Supplement: Online Supplementary Document [file jogh-04-010401-s001.pdf]

Online Supplementary Document

Krezanoski PJ et al. Household malaria knowledge and its association with bednet ownership in settings without large–scale distribution programs: Evidence from rural Madagascar

Journal of Global Health 2014;4:010401

Supplemental table 1: Correlates of household bednet ownership in sample restricted to HOUSEHOLD HEADS (HH)

Variable	Bivariate analysis		Multivariate analysis	
	OR (95% CI)	P value	OR (95% CI)	P value
**Head of household characteristics**				
Age of HH	1.01 (0.98—1.04)	0.453	–	–
Gender of HH	0.79 (0.23—2.67)	0.706	–	–
Education level of HH	1.06 (0.88—1.29)	0.531	–	–
**Household characteristics**				
Number of HH members	1.14 (0.97—1.35)	0.123	3.75 (0.97—14.55)	0.056
Reported fever in last month	3.50 (1.03—11.89)	0.044	1.03 (0.80—1.32)	0.824
Number of children under 5 years	0.90 (0.46—1.75)	0.755	–	–
Number of pregnant women	–	–	–	–
Open water source	1.24 (0.32—4.82)	0.753	–	–
Distance to water source (mns walk)	1.02 (0.95—1.09)	0.567	–	–
**Wealth index**				
...quintile relative to lowest wealth quintile				
Second	–	–	–	–
Third	–	–	–	–
Fourth	0.35 (0.03—3.45)	0.366	0.28 (0.03—3.10)	0.302
Fifth	2.77 (0.67—11.36)	0.158	2.90 (0.47—17.80)	0.251
**Respondent perception/knowledge of malaria**				
Reports fever most frequent illness in village	0.72 (0.15—3.49)	0.684	–	–
Reports fever most dangerous illness in village	1. 41 (0.42—4.74)	0.581	–	–
Malaria knowledge index	4.77 (1.01—22.55)	0.049	5.82 (1.10—30.84)	0.038

N = 250 observations.

CI – confidence interval

Supplemental table 2: Correlates of household bednet ownership in sample restricted to WIVES of household heads

Variable	Bivariate analysis		Multivariate analysis	
	OR (95% CI)	P value	OR (95% CI)	P value
**Head of household characteristics**				
Age of wife	1.01 (0.99—1.04)	0.349	–	–
Gender of wife	–	–	–	–
Education level of wife	1.05 (0.91—1.20)	0.510	–	–
**Household characteristics**				
Number of household members	1.11 (0.97—1.27)	0.139	1.10 (0.95—1.26)	0.200
Reported fever in last month	1.58 (0.74—3.36)	0.234	1.35 (0.61—2.98)	0.458
Number of children under 5 years	1.12 (0.74—1.69)	0.588	–	–
Number of pregnant women	1.72 (0.51—5.84)	0.382	–	–
Open water source	1.12 (0.51—2.45)	0.777	–	–
Distance to water source (mns walk)	1.01 (0.96—1.05)	0.792	–	–
**Wealth index**				
...quintile relative to lowest wealth quintile				
Second	0.34 (0.08—1.35)	0.124	0.43 (0.10—1.78)	0.246
Third	0.72 (0.23—2.24)	0.566	0.92 (0.29—2.98)	0.892
Fourth	0.46 (0.13—1.63)	0.227	0.61 (0.16—2.25)	0.455
Fifth	1.34 (0.48—3.74)	0.572	1.98 (0.66—5.96)	0.221
**Respondent perception/knowledge of malaria**				
Reports fever most frequent illness in village	0.54 (0.19—1.56)	0.254	–	–
Reports fever most dangerous illness in village	1.23 (0.56—2.69)	0.604	–	–
Malaria knowledge index	3.28 (1.41—7.67)	0.006	3.53 (1.44—8.68)	0.006
N = 254 observations unless specified.				

Supplemental table 3: Correlates of household bednet ownership in sample restricted to HOUSEHOLD DECISION MAKERS (Household Heads (HH) or WIVES)

Variable	Bivariate analysis		Multivariate analysis	
	OR (95% CI)	P value	OR (95% CI)	P value
**Head of household characteristics***				
Age of HH	1.01 (0.98—1.05)	0.429	–	–
Gender of HH	0.80 (0.24—2.69)	0.715	–	–
Education level of HH	1.06 (0.87—1.28)	0.559	–	–
**Household characteristics**				
Number of household members	1.15 (1.04—1.27)	0.007	1.10 (0.98—1.24)	0.095
Reported fever in last month	2.21 (1.17—4.17)	0.015	1.82 (0.94—3.54)	0.076
Number of children under 5 years	1.09 (0.77—1.52)	0.638	–	–
Number of pregnant women	1.48 (0.45—4.85)	0.517	–	–
Open water source	1.07 (0.55—2.09)	0.843	–	–
Distance to water source (mns walk)	1.01 (0.97—1.05)	0.652	–	–
**Wealth index**				
...quintile relative to lowest wealth quintile				
Second	1.15 (0.46—4.91)	0.497	1.05 (0.31—3.56)	0.935
Third	1.03 (0.29—3.68)	0.962	0.80 (0.22—2.96)	0.739
Fourth	1.96 (0.63—6.06)	0.244	1.29 (0.39—4.21)	0.675
Fifth	3.8 (1.33—10.73)	0.013	2.68 (0.86—8.34)	0.089
**Respondent perception/knowledge of malaria**				
Reports fever most frequent illness in village	0.66 (0.28—1.56)	0.347	–	–
Reports fever most dangerous illness in village	1.46 (0.77—2.80)	0.249	1.19 (0.60—2.34)	0.620
Malaria knowledge index	3.64 (1.75—7.59)	0.001	3.41 (1.59—7.32)	0.002

N = 560 observations unless specified. *N= 286 because of missing variables.

CI – confidence interval
